# Effect of Composition and Freeze-Thaw on the Network Structure, Porosity and Mechanical Properties of Polyvinyl-Alcohol/Chitosan Hydrogels

**DOI:** 10.3390/gels9050396

**Published:** 2023-05-09

**Authors:** Fernando Soto-Bustamante, Gavino Bassu, Emiliano Fratini, Marco Laurati

**Affiliations:** 1 Dipartimento di Chimica “Ugo Schiff”, Università di Firenze, 50019 Firenze, Italy; 2 Consorzio per lo Sviluppo dei Sistemi a Grande Interfase (CSGI), c/o Università di Firenze, 50019 Firenze, Italy

**Keywords:** hydrogel, cryogel, poly (vinyl alcohol), chitosan, porosity, network structure, rheology

## Abstract

We report the synthesis and characterization of poly (vinyl alcohol) (PVA)/Chitosan (CT) cryogels for applications involving the uptake and entrapment of particulate and bacterial colonies. In particular, we systematically investigated the network and pore structures of the gels as a function of CT content and for different freeze-thaw times, combining Small Angle X-Ray Scattering (SAXS), Scanning Electron Microscopy (SEM), and confocal microscopy. The nanoscale analysis obtained from SAXS shows that while the characteristic correlation length of the network is poorly affected by composition and freeze-thaw time, the characteristic size of heterogeneities associated with PVA crystallites decreases with CT content. SEM investigation evidences a transition to a more homogeneous network structure induced by the incorporation of CT that progressively builds a secondary network around the one formed by PVA. A detailed analysis of confocal microscopy image stacks allows to characterize the 3D porosity of the samples, revealing a significantly asymmetric shape of the pores. While the average volume of single pores increases with increasing CT content, the overall porosity remains almost unchanged as a result of the suppression of smaller pores in the PVA network with the progressive incorporation of the more homogeneous CT network. Increasing the freezing time in the FT cycles also results in a decrease of porosity, which can be associated with a growth in the crosslinking of the network due to PVA crystallization. The linear viscoelastic moduli measured by oscillatory rheology show a qualitatively comparable frequency-dependent response in all cases, with a moderate reduction with increasing CT content. This is attributed to changes in the structure of the strands of the PVA network.

## 1. Introduction

Hydrogels are chemically or physically cross-linked 3D porous polymer networks formed by water-soluble polymers, capable of absorbing a large quantity of water. They find application in several fields, comprising drug delivery [1,2,3], filtration processes [4,5,6], water harvesting [7,8,9], growth of bacterial colonies [10,11,12,13], and cultural heritage restoration [14,15,16]. Different synthetic polymers can be used for producing hydrogels, including poly (ethylene glycol) (PEG) [17], poly (2-hydroxyethyl methacrylate) (PHEMA) [18,19], poly (acrylic acid) (PAA) [20], poly (acrylamide) (PAAm) [21], poly (vinyl alcohol) (PVA) [22,23], among others. In particular, hydrogels formed by PVA have attracted much attention and research effort [24] owing to the non-toxicity of PVA, which is essential in biomedical applications [25], and which combines with a high mechanical strength [26] and a facile tuning of properties [27]. PVA hydrogels are most commonly prepared using physical crosslinking obtained through the freeze-thaw (FT) method first introduced by Peppas and coworkers [28]. In the freezing step, the formation of ice crystals favors the segregation of PVA-rich phases, in which polymer–polymer interactions facilitate hydrogen bonding and crystallization. The so-formed PVA crystallites constitute the physical junctions of the polymer network. In the thawing step, the ice crystals act as porogens and create the pores of the network. Repetition of FT cycles drags an increasingly larger amount of PVA into the network structure and leads to stronger gels [29]. Due to the coexistence of crystallization and phase separation, the duration of the freeze and thaw steps, temperature, molecular weight, concentration and hydrolysis degree of PVA also influence the gel properties. Recent emerging applications of PVA hydrogels obtained by FT include actuators [30] and sensors [31], photonic crystals [32], art restoration [16], and artificial tissue [33].

In view of the growing concerns regarding the sustainability of synthetic polymers, research efforts increasingly focused on the development of hydrogels based on biopolymers from natural sources, in particular natural polysaccharides [34,35]. Chitin is the second most abundant polysaccharide that can be found in nature after cellulose. It can be isolated from the shells of crabs and shrimps, from insect cuticles, and from fungal biomass [36]. Chitosan (CT), obtained from chitin deacetylation or from the enzymatic treatment of chitin deacetylase, is therefore an abundant, biocompatible, non-toxic and biodegradable polysaccharide with important biological functions such as antioxidant, antimicrobial, anticancer, anti-inflammatory, hemo-compatible, and hemostatic activities [37,38,39], which have been exploited in applications such as drug delivery [40], wound healing [41], tissue engineering [42], and preservation of food [43]. CT hydrogels, which are formed by physical association [44] and through complexation with anionic molecules [45] or polyelectrolites [46], unfortunately typically suffer from poor mechanical properties. However, due to the presence of functional amino and hydroxil groups, CT can be easily modified and blended with other copolymers that can contribute to the mechanical stability of the network, including PVA. Preparation of PVA/CT composite hydrogels through freeze-thaw has been already reported, especially in the framework of biomedical applications [47,48], in particular related to: drug delivery [49], wound healing [50,51], and the production of scaffold materials [52]. These studies focused mainly on aspects related to the material application and reported partial characterization of the microscopic properties of this class of hydrogels. Therefore, a systematic study of the link between composition, freeze-thaw, and physical properties of the hydrogels, such as the network microstructure and the mechanical response, is lacking.

In this work, we combine complementary characterization techniques that cover a broad range of length scales going from the nano- to the microscale, including small-angle X-ray scattering (SAXS), scanning electron microscopy (SEM), and confocal laser scanning microscopy (CLSM), to report a systematic investigation of the structural properties of PVA/CT hydrogels with different relative concentrations of the two components, and obtained through FT cycles presenting different relative duration of freeze and thaw. We additionally explore the effects of CT molecular weight. Using a detailed analysis of the scattering and microscopy experiments, we extract a complete set of descriptors characterizing the network conformation, its heterogeneity and the 3D pore structure. We complete our study through rheological measurements of the linear viscoelasticity of the hydrogels, which is linked to the structural properties. The reported characterizations are particularly relevant for the understanding of transport phenomena in these hydrogels, such as uptake and trapping of particulate or bacterial colonies.

## 2. Results and Discussion

### 2.1. Swelling Behavior

**Figure 1:**
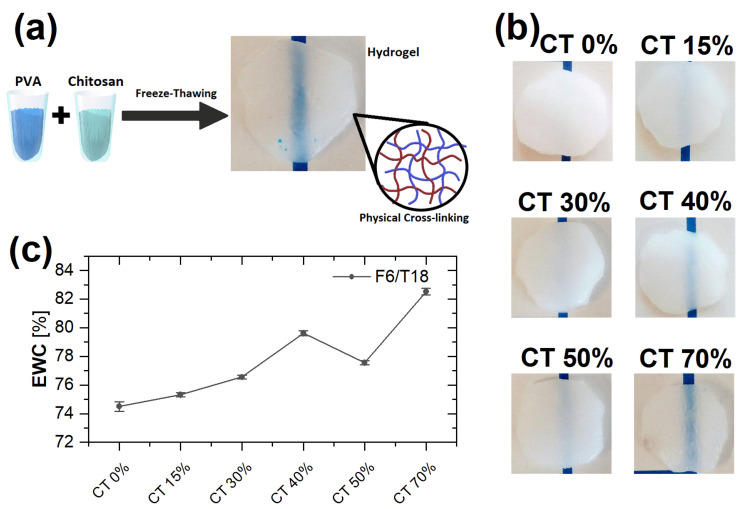
(**a**) Schematic representation of the preparation of hydrogels formed from PVA and CT using physical crosslinking. (**b**) Pictures of the corresponding hydrogels on a paper substrate. The blue line on the substrate is used to indicate the degree of transparency. (**c**) Equilibrium water content (EWC) as a function of CT content, for the freeze-thaw cycle F6/T18.

Figure 1b reports pictures of the swollen hydrogels synthesized with different amounts of CT using a freeze-thaw cycle F6/T18. The maximum CT content in the hydrogels is 70% volume since for larger values, the gels were found to be mechanically unstable.

It can be noticed that while the pure PVA gel is relatively opaque, due to the presence of PVA crystallites and of a heterogeneous network, for increasing CT content, the hydrogels become increasingly transparent. This suggests that the structure becomes less heterogeneous and that crystallinity is reduced with increasing CT content. The (c) panel of Figure 1 shows EWC values obtained for the same hydrogels of Figure 1b. For all samples, a pronounced hydration is observed. EWC values indicate for F6T18 and F12T12, a smooth increase of hydration as a function of CT content, while for F18T6, there is an initial increase followed by a decrease for CT content > 40% (data for additional FT cycles in the Supplementary Information). For the shortest freezing time (6 h, data in Figure 1), the maximum EWC is obtained for 70% CT content. For the FT cycle F12/T12, the variation of hydration with composition is moderate and with maximum values at 40% and 70% CT content (Supplementary Information). Finally, for F18/T6, the maximum of EWC is observed for 40% CT content (Supplementary Information). In addition, the hydration values slightly increase with increasing freezing time for CT content < 40%.

### 2.2. Nanoscale Structure: SAXS

**Figure 2:**
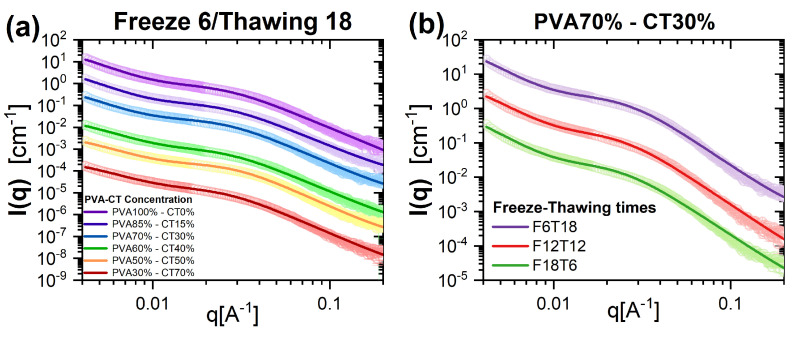
SAXS intensity curves I(q) vs. *q* for (**a**) samples with different PVA/CT content obtained using the same FT cycle (6 h/18 h), and (**b**) samples with PVA/CT content 70%/30% and different FT cycles. For clarity, curves have been vertically shifted on the y-axis with respect to the pure PVA sample. Lines are model fits according to Equation (1).

SAXS intensities I(q) measured for samples with different compositions (Figure 2a) and FT cycles (Figure 2b) present a qualitatively comparable *q*-dependence, characterized by an increase of I(q) at low *q* values, indicating that larger structures are present, an intermediate plateau region, and a decay for q≳0.05 Å${}^{-1}$. The experimental data were modeled using a generalized version of the Debye–Bueche function [53], which is used to model the structure of fractal gel networks. This model is described by the following expression [54]:

(1) $$I\left(q\right)=I_{\mathrm{sol}}\left(q\right)+I_{\mathrm{ex}}\left(q\right)+bkg$$

The first term corresponds to the generalized version of the Ornstein–Zernike model [55]:

(2) $$I_{\mathrm{sol}}\left(q\right)=\frac{I_{\mathrm{lor}}\left(0\right)}{1+{\left(\xi q\right)}^{m}}$$

which is used to describe the homogeneous scattering from polymer network structures with characteristic correlation length ξ. The quantity $I_{\mathrm{lor}}\left(0\right)$ represents the scattering intensity at q=0, which is determined by the contrast between the polymer and the solvent, and by the volume fraction of the polymer within the gel. The parameter *m* is the Porod exponent, reflecting the interactions between the polymer and the solvent. For linear polymer chains in a good solvent, m=1.67, while *m* progressively increases with the worsening of the solvent conditions and the associated polymer collapse [56].

The second term in the Debye–Bueche function is used to model the forward scattering at low *q*, arising from solid-like heterogeneities of average size *a* [57]:

(3) $$I_{\mathrm{ex}}\left(q\right)=\frac{I_{\mathrm{ex}}\left(0\right)}{{\left(1+a^{2}q^{2}\right)}^{2}}$$

in which Iex(0) is the excess scattering at q=0, which is again related to the contrast and the volume fraction of the heterogeneities. The last term bkg describes a constant background.

**Figure 3:**
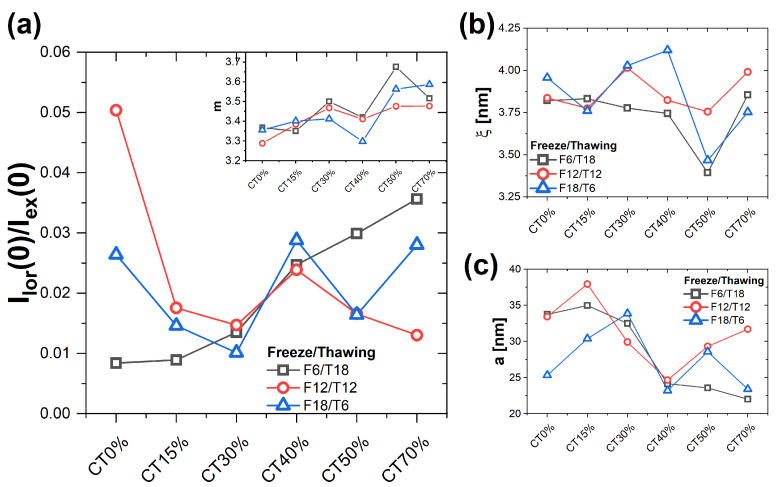
Dependence on CT content of the following parameters obtained by modeling the experimental I(q) using Equation (1), for different values of FT times: (**a**) Ratio between the prefactors of the network (Equation (2)) and solid-like heterogeneities (Equation (3)) contributions to the total scattering, $I_{\mathrm{lor}}\left(0\right)/I_{\mathrm{ex}}\left(0\right)$. Inset: exponent *m* of the network contribution. (**b**) Correlation length ξ. (**c**) Average size of the heterogeneities, *a*.

Figure 3 shows the trends of the most representative parameters obtained from the fitting of the model, as a function of gel composition and for different FT cycles. Panel (a) reports the values of the ratio $I_{\mathrm{lor}}\left(0\right)/I_{\mathrm{ex}}\left(0\right)$, which is used to evaluate the relative contribution of the scattering arising from the fractal network and from the solid-like heterogeneities, associated with the respective volume fractions of polymer in the network and solid-like heterogeneities. For all compositions and for the different FT cycles, the scattering from heterogeneities dominates. For F6/T18, the ratio increases with increasing CT content, which could be interpreted as a reduction of the volume fraction of solid-like heterogeneities compared to polymers in the network. If one excludes the pure PVA gel sample (CT0%), for F12/T12, the ratio shows moderate variations with a maximum at 40% CT content. Finally, for F18/T6, again excluding the pure PVA sample, the data indicate an overall slight increase with increasing CT, with the presence of fluctuations and a maximum at 40% CT content. An increase in the ratio $I_{\mathrm{lor}}\left(0\right)/I_{\mathrm{ex}}\left(0\right)$ could be interpreted as an indication of a slightly more homogeneous network, resulting from the lower fraction of PVA and therefore of crystalline regions. For F6T18, this interpretation is coherent with the increase in transparency and with the trend of the EWC.

The fractal exponents *m* (Figure 3a, inset) are all larger than 3, indicating a large degree of collapse of the polymers in the network. The values of *m* slightly increase with increasing CT content, in particular for the shortest and longest freezing times, similar to what was found for $I_{\mathrm{lor}}\left(0\right)/I_{\mathrm{ex}}\left(0\right)$. No clear trend with FT can be extracted in this case. The increase in *m*, even if moderate, seems to suggest that the mixing of the two polymers induces additional segregation between the two species and a more globular configuration of the coils. The correlation length ξ (Figure 3b) is in all cases between 3.2 nm and 4.2 nm. For the intermediate freezing time (12 h), the variation is moderate, while for the other FT cycles, the quantity presents a minimum for 50% CT content. For a freezing time of 18 h, there is also a maximum observed at 40% CT content. However, also in these cases, the relative changes are small. Therefore, the correlation length of the network does not seem to be strongly affected by composition nor by the FT cycle. The average size *a* of the heterogeneities (Figure 3c) presents in all cases a decrease when increasing CT content above 15%, starting from values around 30 nm and decreasing down to about 20 nm. For the cycle F12/T12, the value starts to increase again for CT content > 50%. The decrease of the *a* value suggests that an increase in CT content results in more homogeneous gel networks at the mesoscopic scale, in agreement with what commented regarding the ratio $I_{\mathrm{lor}}\left(0\right)/I_{\mathrm{ex}}\left(0\right)$.

### 2.3. Microscale Porosity: Confocal Microscopy and SEM

**Figure 4:**
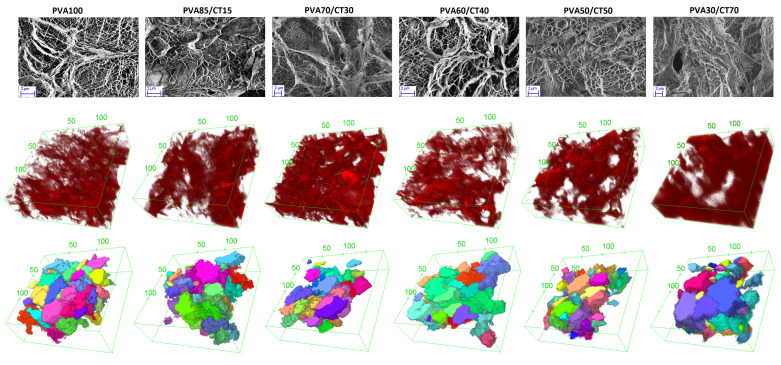
Characterization of the microscale porosity of gels obtained with a F6/T18 cycle and different PVA/CT content. Top: SEM images of selected gel sections. Middle: 3D stacks from confocal microscopy experiments on fluorescent-labeled gels. Bottom: Renderings of the pore reconstruction obtained using MorpholibJ. For the 3D stacks and renderings, the units on the boxes’ axes are μm.

The 2D and 3D porous structure of the gels was analyzed at the microscale using a combination of SEM and confocal microscopy. Figure 4 shows exemplary results obtained for F6/T18 and different PVA/CT compositions. Corresponding analyses for samples obtained with other FT cycles are reported in the Supplementary Information. The top row of Figure 4 presents representative images obtained by SEM. These show for all samples a disordered porous structure with a large distribution of characteristic pore sizes. The pure PVA gel shows a well-defined network structure characterized by interconnected strands, some of them being particularly thick. The hydrogel with 15% CT content has a similar strand structure; however, a small number of more homogeneous regions is present. The sizes of the pores in these samples is predominantly at the microscale or larger. Further increasing CT content to 30%, the presence of smaller pores becomes evident, together with a larger fraction of more homogeneous regions. These regions are therefore expected to be mainly composed of CT. In the sample with 40% CT content, the smaller porosity is still present, while the homogeneous regions seem to surround the thicker strands. For 50% CT content, the more homogeneous network is considerably more extended and the smallest pores start to disappear. This tendency is even clearer for the sample with 70% CT content, where the more homogeneous regions extend over the whole imaged region of the hydrogel. Note also that for the samples with CT content 50% and 70%, the thickness of the PVA strands reduces.

The heterogeneous and disordered porous structure at the microscale (and larger) is visualized in 3D through the stacks obtained with confocal microscopy (Figure 4, mid- row). While a clear dependence of pore size and density on CT content cannot be clearly evinced from single image stacks, the ones corresponding to 30% and 70% CT content present a smaller fraction of large pores, a finding that is consistent with the results of SEM. These observations are confirmed by the 3D analysis of the porosity performed using MorpholibJ, the results of which are reported on the bottom row of Figure 4. In the renderings, different pores obtained from the analysis are distinguished using different colors. The renderings also confirm the broad distribution of pore sizes.

**Figure 5:**
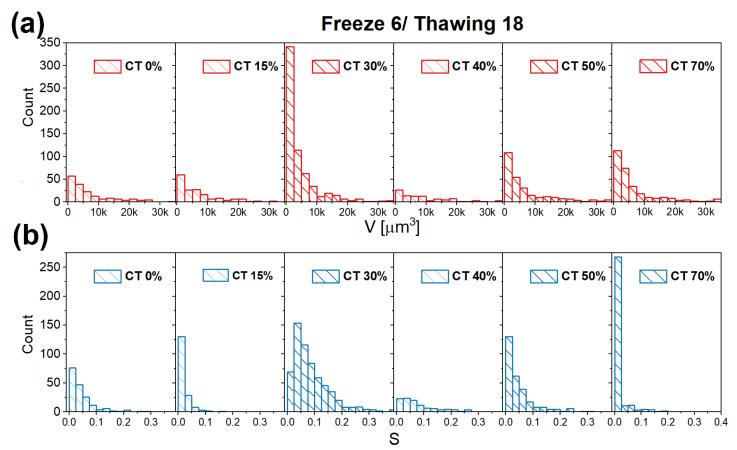
(**a**) Pore volumes distribution and (**b**) Sphericity (S) distribution for samples obtained with a F6/T18 cycle and having different CT content.

The distributions of pore volumes and sphericity were determined for the different samples by analyzing a set of stacks. Figure 5 shows the distributions obtained for the samples of Figure 4, additional distributions determined for samples obtained using different FT cycles are reported in the SI. The distributions of pore volumes confirm the qualitative discussion of the stacks reported in Figure 4: their distributions are particularly broad and extend to larger volumes for the pure PVA sample and for samples with CT content 15% and 40%, while they become shifted towards smaller values for CT contents of 30%, 50%, and 70%. The sphericity index distributions show that the pores are considerably elongated. Samples with CT contents of 30% and 50% present distributions that extend to larger sphericity values.

**Figure 6:**
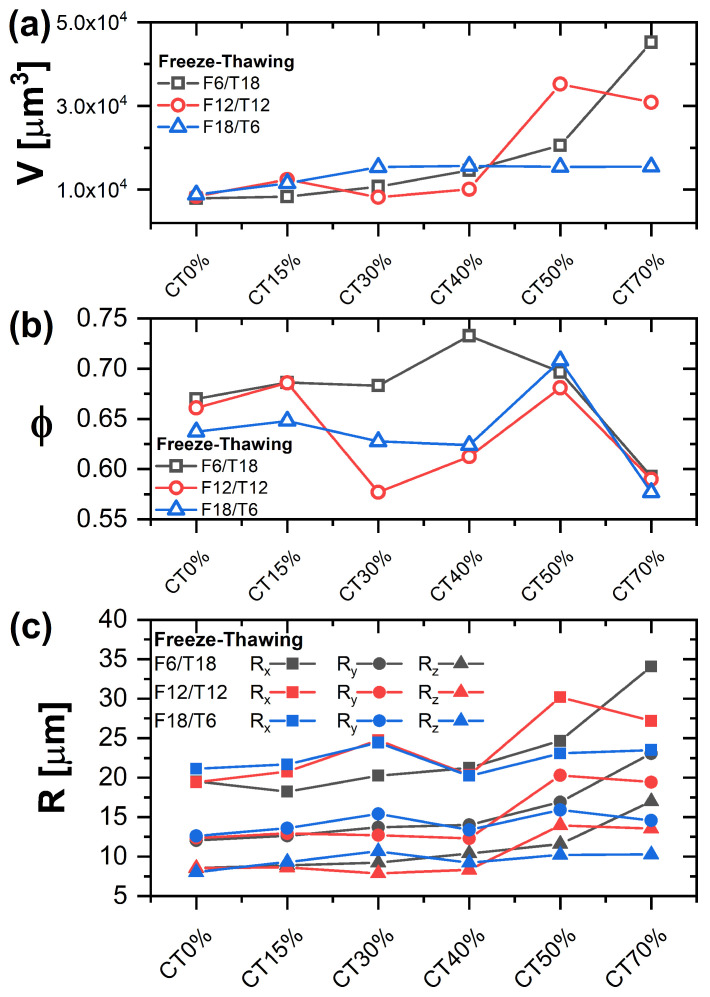
Porosity parameters as a function of PVA/CT content, for different FT cycles. (**a**) Average pore volume. (**b**) Porosity, (**c**) size of the axes of the pore filling ellipsoids, R${}_{x}$, R${}_{y}$, R${}_{z}$.

Mean parameter values where extracted from the distributions and are reported in Figure 6. For samples obtained with F6/T18 and F12/T12 cycles, the average pore volume (Panel a) is increasing with increasing CT content, i.e., larger pores are formed on average, in particular for CT contents larger than 40%. On the other hand, after a slight increase up to a CT content of 30%, the average pore volume remains essentially unchanged for F18/T6. This result confirms the presence of important effects associated with the parameters of the FT cycle. Note also that the observed trends of the average pore volume are coherent with those of the EWC. Panel c reports the average sizes $R_{i}$ of the ellipsoid axes filling the pore volumes, being *i* a Cartesian coordinate, as a function of CT content. These values allow to determine the symmetry of the pores and, in our case, show that the pores are asymmetric, as also suggested by the sphericity distributions in Figure 5. The pores present the average largest size in the x-direction (R${}_{x}$). Therefore, they are elongated in the x-y plane, with R${}_{x}$ being almost double R${}_{y}$, and relatively flat in *z*, with R${}_{z}$ being the smallest size (about half of R${}_{y}$). The increase of pore volume with CT content observed in panel A is associated with an increase in all 3 characteristic sizes for F6/T18 and F12/T12. While the average pore volume increases for F6/T18 and F12/T12, the porosity presents a different trend, indicating that the increase in the volume is not accompanied by a corresponding increase in the number of pores. For F6/T18, the maximum porosity, ϕ≈0.73, is observed for 40% CT content, while it significantly decreases for larger CT amounts, being about 0.60 for 70% content. For F12/T12, the values of ϕ for the smallest and largest CT contents are comparable to those of F6T18, while they are significantly smaller for intermediate CT contents, presenting a minimum value ϕ≈0.57 for 30% CT content. Finally, for F18T6, the porosity at low and intermediate CT contents is almost constant and about 0.63; therefore, it is smaller than for F6T18, while for CT ≳ 50%, the values are again comparable to those found for the other FT cycles. These results suggest that in samples with larger PVA content, the degree of porosity can be better tuned by changing FT parameters, possibly as a result of the effect on the degree of crystallization of the PVA component.

### 2.4. Mechanical Properties: Rheology

**Figure 7:**
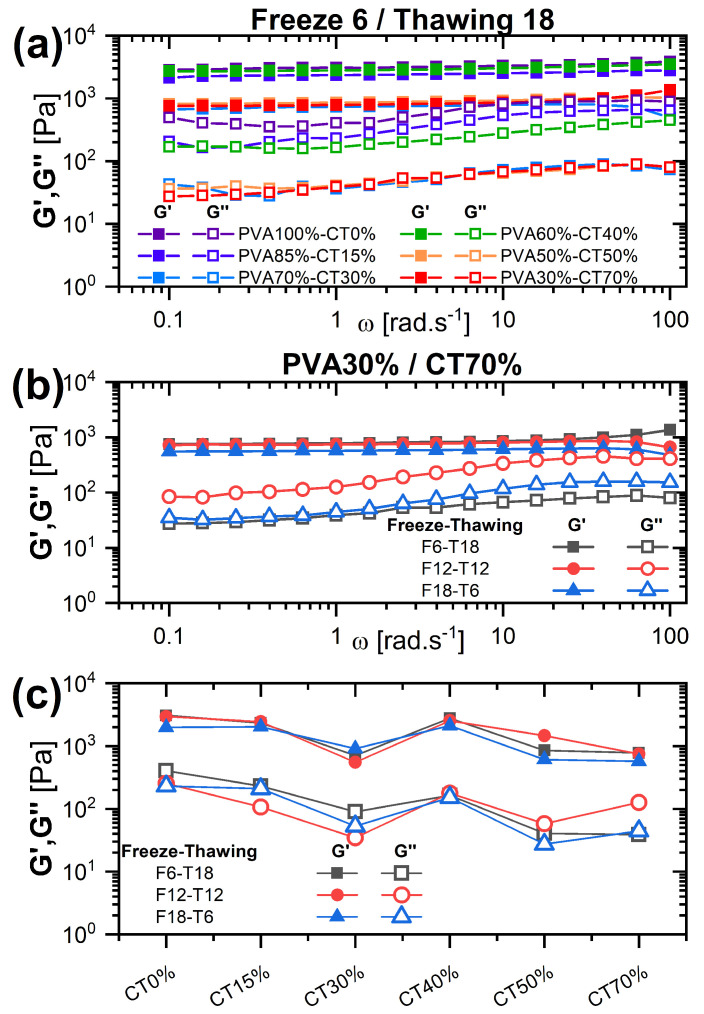
Frequency-dependent linear viscoelastic moduli of hydrogels with different PVA/CT content and for distinct FT cycles. (**a**) G${}^{\prime }\left(\omega \right)$, G${}^{\prime \prime }\left(\omega \right)$ for F6/T18 and various PVA/CT contents. (**b**) G${}^{\prime }\left(\omega \right)$, G${}^{\prime \prime }\left(\omega \right)$ for PVA30%/CT70% and different FT cycles. (**c**) Storage and loss moduli extracted at ω=1 rad/s, as a function of CT content, for different FT cycles.

We explored the effects of the structure and porosity of the network on the mechanical properties of the hydrogels by determining their frequency-dependent linear viscoelastic moduli using oscillatory rheology. Figure 7a shows exemplary data obtained for the FT cycle F6/T18 and the different PVA/CT contents investigated. As expected, all samples present a solid-like response, with G${}^{\prime }\left(\omega \right)$ > G${}^{\prime \prime }\left(\omega \right)$ for all frequencies and an almost frequency-independent G${}^{\prime }\left(\omega \right)$. A reduction in the moduli is observed when comparing the pure PVA gel and the one with the largest CT content; however, the trend for intermediate values is non-monotonic. This is better visualized in Figure 7c, in which G${}^{\prime }(\omega =1$ rad/s) and G${}^{\prime \prime }(\omega =1$ rad/s) are reported as a function of CT content. Both moduli decrease initially up to 30% CT content, then increase for 40% CT, and finally decrease again for larger CT contents. Increasing the freezing time and correspondingly reducing the thawing time is mainly affecting the loss modulus G${}^{\prime \prime }\left(\omega \right)$, which becomes more frequency-dependent and approaches the storage modulus, suggesting a certain degree of softening of the structure (Figure 7b). However, the effect is not particularly pronounced, as indicated by the comparison between all compositions and FT cycles in Figure 7c.

### 2.5. Effect of Chitosan Molecular Weight

**Figure 8:**
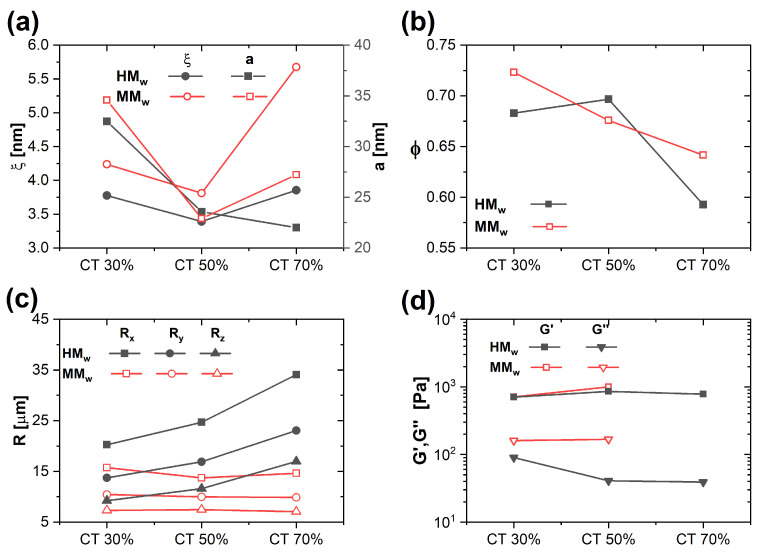
Effect of CT M${}_{w}$ on the structure, porosity, and rheology of hydrogels. In all plots, parameters obtained for high M${}_{w}$ CT are compared to the corresponding ones obtained for medium M${}_{w}$ CT (**a**) ξ and *a* from SAXS, (**b**) porosity ϕ, and (**c**) pore dimensions from confocal microscopy, (**d**) linear viscoelastic moduli.

The effect of CT M${}_{w}$ on the structure, porosity, and mechanical properties of the hydrogels was determined by comparing the results of characterizations obtained for samples containing CT of high and medium M${}_{w}$ (Figure 8, for F6/T18). Panel (a) reports a comparison of the parameters ξ and *a* obtained from the analysis of SAXS data. While the average size of heterogeneities *a* is comparable for the two M${}_{w}$, the correlation length ξ increases with decreasing M${}_{w}$. This might be attributed to a lower degree of entanglement of the CT network for smaller M${}_{w}$. The porosity extracted from the analysis of confocal microscopy image stacks shows comparable values for the two molecular weights. However, the values of *R* (Panel c) for the medium M${}_{w}$ show much less dependence on CT content, while maintaining the same kind of asymmetry, with R${}_{x}$ > R${}_{y}$ > R${}_{z}$. The smaller dependence on CT suggests that the structure of the PVA network is less influenced by the lower M${}_{w}$ CT. Finally, while the storage moduli are comparable for the two M${}_{w}$, the loss moduli are larger for samples containing the medium molecular weight CT. The smaller difference between G${}^{\prime }$ and G${}^{\prime \prime }$ for the medium M${}_{w}$ indicates a more pronounced fluid-like response of the samples, which is consistent with a looser network and a larger mesh size ξ. Note that for the medium M${}_{w}$, the sample with CT content 70% was too brittle to be measured in the rheometer.

## 3. Conclusions

Motivated by the development of hydrogels containing a significant fraction of a natural polysaccharide, in this work, we investigated variations in the swelling behavior, network and porous structure, and mechanical properties of hydrogels formed by mixtures of PVA and CT with different relative compositions and using distinct freeze-thaw cycles. For samples obtained with freeze-thaw cycles F6T18 and F12T12, we found that with increasing CT content, the degree of hydration moderately increases, while for F18T6, it increases initially and starts to decrease for CT content > 40%. Moreover, EWC values are larger for larger freezing times at small CT contents, and vice versa for large CT contents. These trends are coherent with the variation of the average pore volume obtained from the confocal microscopy analysis of pore structures. SEM images show that the network structure of PVA is progressively altered by mixing with increasing amounts of CT. In particular, PVA strands get progressively thinner, porosity at sub-micron scales develops, and the PVA network is progressively surrounded by a more homogeneous network of CT.

At the nanoscale, where the internal properties of strands are observed, SAXS measurements were described in terms of contributions from a polymer network and from solid-like heterogeneities, which mainly represent crystallites of PVA. Increasing the CT content, the contribution of heterogeneities decreases, as well as their average size. This is consistent with the increasing importance of the more homogeneous CT network. The structure of the polymers in the mesh is more compact, indicating possibly a segregation effect, a finding that is again consistent with the presence of two networks in the samples. The characteristic mesh size, however, only shows limited variations with the CT content. There is also no clear influence of the FT times on the network parameters. The analysis of the porous structure at the micron and larger scales evidences the presence of asymmetric pores having volumes that increase with increasing CT content, at least for short freezing times. The overall porosity is only seen to significantly decrease for the largest CT content, and is maximal for short freezing times. Indeed, at the maximum CT content, the presence of the more homogeneous CT network suppresses small size pores, and only a smaller number of larger pores is present. While the frequency dependence of the linear viscoelastic moduli of the gels is comparable for all compositions, with increasing CT content, on average, the magnitude reduces slightly compared to the pure PVA gel. This seems to be associated with the thinning of the PVA strands and to the reduction in the crystallite size. However, at a specific intermediate CT content of 40%, the moduli present values very similar to those of pure PVA. This could be the result of a peculiar structure evidenced by SEM, in which the CT network surrounds PVA strands, leading to a composite network that resembles that of pure PVA.

Our detailed and systematic study of the network and porous structure of the gels, and its relationship with the mechanical response, reveals that the interactions between CT and PVA lead to complex variations of the network structure and to a porosity developing over multiple length scales when varying the relative content of the two polymers. These trends, and those of the related mechanical properties, could not be predicted based on the composition or synthesis procedure alone. Our results will serve as a guide in the preparation of gels for applications that involve transport phenomena, such as bacterial filtration and entrapment, art restoration, and water harvesting.

## 4. Materials and Methods

### 4.1. Chemicals and Synthesis

CT with high ($M_{w}=190–300$ KDa) and medium molecular weight ($M_{w}=50–190$ KDa), 99+% hydrolyzed PVA with $M_{w}=146–186$ KDa, and acetic acid were purchased from Merck (Darmstadt, Germany). All chemicals were used without further purification.

A solution of CT with concentration equal to 2% (*w*/*v*) was obtained by dissolving CT into a 0.1 M solution of acetic acid and milli-Q water (resistivity ρ>18 MΩ· cm) at room temperature for 4 h and under constant vigorous magnetic stirring. A solution of PVA with concentration equal to 5% (*w*/*v*) was prepared in milli-Q water at T=90 °C and mixed for 1 h under constant stirring. The two solutions of CT and PVA were mixed at T=50 °C with the following volume mixing ratios: PVA100%-CT0%, PVA85%-CT15%, PVA70%-CT30%, PVA60%-CT40%, PVA50%-CT50%, and PVA30%-CT70%. The so obtained mixtures were mixed under constant magnetic stirring for 1 h and were subsequently poured into a Petri dish. To obtain physical crosslinking, the samples were frozen at T=−20 °C and thawed at room temperature for 3 cycles, a cycle consisting in a total of 24 h of different freeze-thaw (FT) ratios; the following combinations F6/T18, F12/T12, F18/T6 were tested, where the number after the letter indicates hours. Finally, the formed hydrogels were repeatedly washed with milli-Q water every twelve hours until any residual in the sample was removed [49,51]. A schematic representation of the synthesis procedure is reported in Figure 1a.

### 4.2. Swelling Behavior

Dried hydrogels were immersed in water at room temperature and the water swelling kinetics were followed by measuring the hydrogel weight as a function of immersion time using an analytical balance (Exemplary data in Figure S1 of the Supplementary Information). Before measurement, hydrogels were centrifuged at 1000 rpm for 10 min to drain excess water unconnected into the gel structure. The Equilibrium Water Content (EWC) was calculated as follows:

(4) $$\mathrm{EWC}=\frac{m_{W}-m_{D}}{m_{W}}\times 100$$

where $m_{W}$ and $m_{D}$ are the weighted masses of the swollen and dry gel, respectively. The EWC value was calculated at t=1440 min, when gels reach equilibrium saturation of water.

### 4.3. SAXS

SAXS measurements were performed on a Xeuss 3.0 HR (Xenocs, Grenoble, France ) equipped with an EIGER2R (1 M model) hybrid pixel photon counting detector (Dectris Ltd., Baden, Switzerland) consisting of 1028 × 1062 pixels with a size of 75 × 75 ${\mu m}^{2}$. The wavelength of the X-ray beam was λ = 1.542 Å. Calibration of the sample to detector distance was performed using silver behenate (d = 58.38 Å). The instrument’s sample chamber was maintained at atmospheric pressure to minimize water evaporation from the samples. Two sample-to-detector distances, 500 and 1800 mm, were used to access a range of scattering vectors *q* going from 0.004 to 0.2 Å${}^{-1}$, with q=(4π/λ)sinθ and 2θ the scattering angle. Hydrogels were enclosed in sealed demountable cells using Kapton^®^ foils as windows. Absolute scattering intensities in (mm${}^{-1}$) were obtained by using glassy carbon as a secondary standard. The 1D azimuthally averaged scattering patterns were reduced by subtracting the scattering intensity from empty holder + water and by merging the curves obtained at the two sample-to-detector distances. Data reduction, normalization, and merging was performed in XSACT (X-ray Scattering Analysis and Calculation Tool, Xenocs, France).

### 4.4. Confocal and Scanning Electron Microscopy

Confocal microscopy imaging was carried out on fluorescently labeled hydrogel samples, fully hydrated, by using a Leica TCS SP8 confocal microscope. Samples were contained in Lab-Tek Chambered Coverglass with a 1.0 borosilicate glass coverslip at the bottom, and imaged using a 63× oil immersion objective having N.A. = 1.43. A 561 nm wavelength laser was used to excite Rhodamine B (RhB) and the fluorescence emission was collected through a highly efficient hybrid detector in the 571–600 nm range. 3D image stacks of 512 × 512 × 251 pixels${}^{3}$, which correspond to hydrogel volumes of 184 × 184 × 50 ${\mu m}^{3}$, were acquired in about 260 s. SEM measurements of the xerogels were obtained using a FEG-SEM ΣIGMA (Carl Zeiss, Aalen, Germany) with an acceleration potential of 2 kV and a working distance of about 3 mm. Under these conditions, metallization of the samples was not necessary.

### 4.5. Image Analysis to Characterize the Porous Structure

Image stacks obtained from confocal microscopy were processed and analyzed using MorphoLibJ, an ImageJ plugin that allows the characterization of the 3D structures of pores in the hydrogels [58]. Once the pores were determined in MorphoLibJ, their volume was calculated and used to obtain the pore volume fraction $\phi =V_{\mathrm{pores}}/V_{\mathrm{tot}}$ of the hydrogel, where $V_{\mathrm{pores}}$ is the cumulative volume occupied by pores over the total volume of the hydrogel, $V_{\mathrm{tot}}$. This quantity is often reported as porosity in the literature. Pores on the edges of the volume were removed to avoid bias on the geometrical data processing. The tool “analyze regions 3D” of MorphoLibJ additionally allowed the investigation of pore morphology and symmetry. Morphological information was obtained through the normalized sphericity index, $\mathrm{SI}=36\pi V^{2}/A^{3}$, where *V* is the sample volume and *A* the surface area for each sample. Regarding symmetry, the size and the orientation of each inertia ellipsoid that best fits within a pore was extracted from the analysis, being R${}_{x}$, R${}_{y}$, and R${}_{z}$ the three characteristic axes of the ellipsoid in order of decreasing magnitude. In addition to the 3D characterization of the pore structure, also 2D sections were analyzed for comparison, extracting the characteristic areas, circularity, and area fraction of pore sections (results in the Supporting Information).

### 4.6. Rheology

Rheological measurements were performed on a DHR-3 hybrid rheometer (TA Instruments, Milano, Italy) using a 20 mm aluminum plate-plate geometry. Hydrogel discs were squeezed between the plates and measurements were performed maintaining a constant normal force of 0.3 N. Dynamic Frequency sweeps were performed for all samples applying a strain amplitude $\gamma_{0}=1$% at oscillation frequencies in the range 0.1≤ω≤100 rad/s.

## Data Availability

The data presented in this study are available on request from the corresponding author.

## Supplementary Materials

The following supporting information can be downloaded at: https://www.mdpi.com/article/10.3390/gels9050396/s1, Figure S1: Additional data on the swelling behavior for F12T12 and F18T6; Figure S2: Additional SAXS data for F12T12 and F18T6; Figure S3: Additional SAXS data for medium M${}_{w}$ CT. Figure S4: Additional SEM, confocal microscopy data and pore reconstructions obtained for samples with composition PVA30%–CT70% and FT cycles F12T12, F18T6; Figure S5: Additional SEM, confocal microscopy data and pore reconstructions obtained for samples with composition PVA50%–CT50% and FT cycles F12T12, F18T6; Figure S6: Additional SEM, confocal microscopy data and pore reconstructions obtained for samples with composition PVA70%–CT30% and FT cycles F12T12, F18T6; Figure S7: 2D porosity parameters; Figure S8: Pore volume and sphericity distributions for F12T12; Figure S9: Pore volume and sphericity distributions for F18T6; Figure S10: Additional rheological data for F12T12 and F18T6; Table S1: Fit parameters obtained from modeling SAXS curves of high M${}_{w}$ CT hydrogels; Table S2: Fit parameters obtained from modeling SAXS curves of medium M${}_{w}$ CT hydrogels.

## Abbreviations

The following abbreviations are used in this manuscript:

PVA	Poly-vinyl-Alcohol
CT	Chitosan
FT	Freeze-Thaw
SAXS	Small Angle X-Ray Scattering
SEM	Scanning Electron Microscopy
