# Supplementary material for: Effect of Composition and Freeze-Thaw on the Network Structure, Porosity and Mechanical Properties of Polyvinyl-Alcohol/Chitosan Hydrogels
Source: Gels. 2023 May 9;9(5):396. doi: 10.3390/gels9050396 (PMC10217673; doi:10.3390/gels9050396)
Supplement: Supplementary file 1 [file gels-09-00396-s001.zip › gels-2347040-supplementary.pdf]

Supplementary Information

Effect of composition and freeze-thaw on the network structure, porosity and mechanical properties of Polyvinyl-alcohol/Chitosan hydrogels

Fernando Soto-Bustamante[1,2], Gavino Bassu[1,2], Emiliano Fratini[1,2] and Marco Laurati[1,2]*
[1] Dipartimento di Chimica, Università di Firenze, 50019 Sesto Fiorentino, Italy
[2] Consorzio per lo Sviluppo dei Sistemi a Grande Interfase (CSGI), c/o Università di Firenze, 50019 Sesto Fiorentino, Italy
*Correspondence: marco.laurati@unifi.it

We report additional experimental data and extracted parameters that complement those presented in the main article. Section 1 reports EWC values for gels obtained with FT cycles F12/T12 and F18T6, and exemplary kinetic curves used to determine EWC, for gels obtained with F6T18. Section2 presents additional SAXS measurements and the corresponding parameters obtained from modeling for: i) samples containing high M_w Chitosan and obtained through Freeze-Thaw cycles F6/T18 and F18/T6, ii) samples containing medium M_w Chitosan and obtained through Freeze-Thaw cycles F6/T18, F12/T12 and F18/T6. Section 3 reports a comparison of SEM images, confocal stacks and porosity renderings of samples containing high M_w Chitosan and obtained through Freeze-Thaw cycles F6/T18, F12/T12 and F18/T6. Section 4 presents i) plots of the parameters characterizing the 2D porosity of samples containing high M_w Chitosan. ii) plots of the distributions of pore volumes and sphericity of samples containing high M_w Chitosan and obtained through Freeze-Thaw cycles F12/T12 and F18/T6. Section 5 reports linear viscoelastic moduli obtained from oscillatory rheology of samples containing high M_w Chitosan and obtained through Freeze-Thaw cycles F12/T12 and F18/T6

1. Swelling behavior

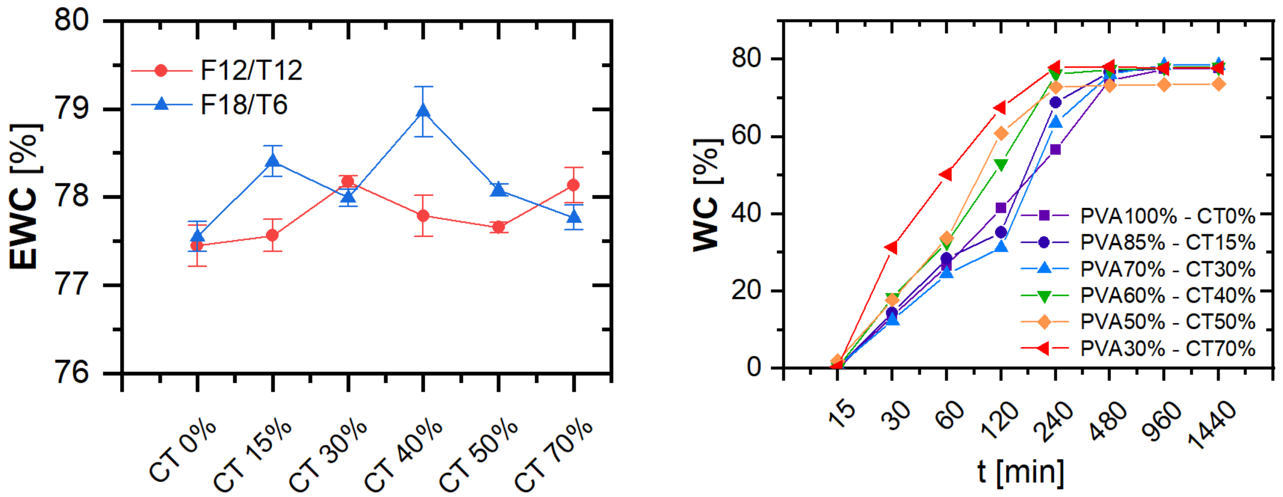

Figure S1. Left: Equilibrium water content (EWC) for the freeze-thawing cycles F12/T12 and F18/T6 as a function of Chitosan content. To drain excess water unconnected into the gel structure, all hydrogels were centrifuged at 1000 rpm for 10 minutes. Right: Exemplary kinetic adsorption curves used to determine EWC, for gels obtained with F6T18.

2. Nanoscale structure, SAXS parameters and plots

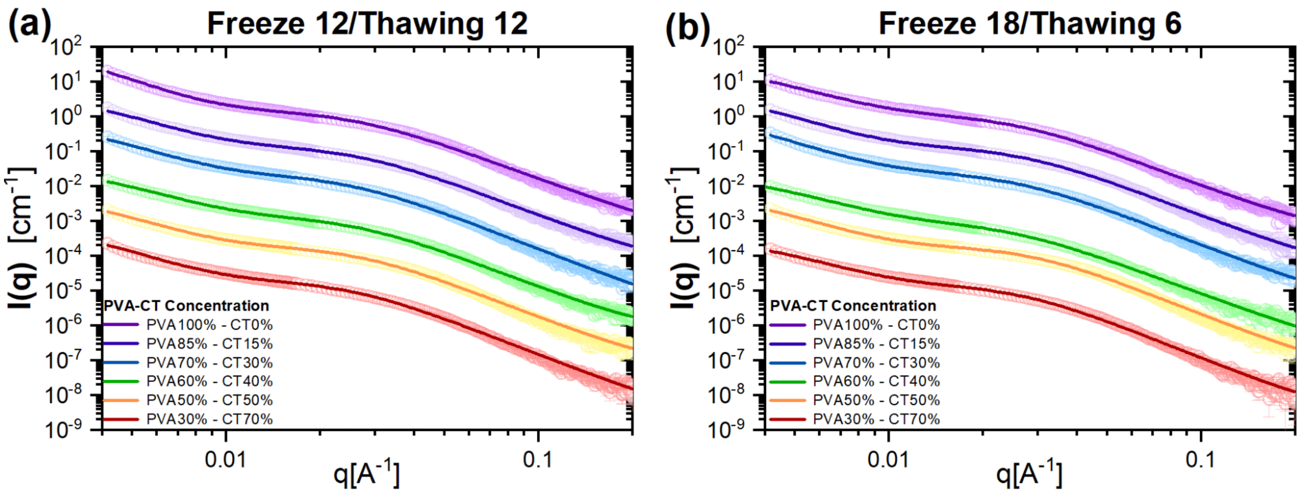

Figure S2. SAXS intensity curves I(q) vs. q for samples with different PVA/CT content obtained using the same FT cycle **(a)** F12/T12 and **(b)** F18/T6. For clarity the curves are displaced along the y-axes. Lines are fits to the model of eq. 3 in the main article

Table S1. SAXS parameters obtained from modeling the curves reported in figure S1 with equation 3 of the main article.

Sample	Ilor(0)	ζ [nm]	m	Iex(0)	a [nm]
P100%-C0% F6/T18	0.86 ± 0.01	3.8209 ± 0.01	3.37 ± 0.02	101.89 ± 0.84	33.75 ± 0.20
P100%-C0% F12/T12	1.38 ± 0.01	3.84 ± 0.01	3.29 ± 0.02	27.45 ± 0.73	33.41 ± 0.18
P100%-C0% F18/T6	1.05 ± 0.01	3.96 ± 0.01	3.36 ± 0.02	39.76 ± 0.69	25.32 ± 0.17
P85%-C15% F6/T18	1.25 ± 0.01	3.83 ± 0.02	3.36 ± 0.02	139.61 ± 0.97	34.97 ± 0.24
P85%-C15% F12/T12	1.32 ± 0.01	3.77 ± 0.02	3.38 ± 0.02	75.238 ± 0.34	37.93 ± 0.18
P85%-C15% F18/T6	1.30 ± 0.01	3.76 ± 0.02	3.40 ± 0.02	89.038 ± 0.49	30.37 ± 0.18
P70%-C30% F6/T18	2.28 ± 0.01	3.78 ± 0.01	3.50 ± 0.01	168.65 ± 0.32	32.49 ± 0.02
P70%-C30% F12/T12	1.96 ± 0.01	4.01 ± 0.01	3.46 ± 0.01	133 ± 0.22	29.92 ± 0.02
P70%-C30% F18/T6	2.41 ± 0.01	4.03 ± 0.01	3.41 ± 0.01	237.34 ± 0.40	33.88 ± 0.02
P60%-C40% F6/T18	1.04 ± 0.01	3.74 ± 0.02	3.42 ± 0.02	42.06 ± 0.54	24.15 ± 0.12
P60%-C40% F12/T12	1.25 ± 0.01	3.82 ± 0.02	3.41 ± 0.02	52.28 ± 0.69	24.67 ± 0.13
P60%-C40% F18/T6	0.87 ± 0.01	4.12 ± 0.01	3.30 ± 0.02	30.37 ± 0.51	23.21 ± 0.16
P50%-C50% F6/T18	2.09 ± 0.01	3.39 ± 0.01	3.68 ± 0.01	69.92 ± 0.12	23.58 ± 0.01
P50%-C50% F12/T12	1.75 ± 0.01	3.75 ± 0.01	3.48 ± 0.01	105.66 ± 0.21	29.32 ± 0.02
P50%-C50% F18/T6_3	1.77 ± 0.01	3.47 ± 0.01	3.56 ± 0.01	107.87 ± 0.67	28.57 ± 0.12
P30%-C70% F6/T18	1.60 ± 0.01	3.85 ± 0.01	3.52 ± 0.01	44.91 ± 0.08	22.01 ± 0.02
P30%-C70% F12/T12	1.83 ± 0.01	3.99 ± 0.01	3.48 ± 0.01	140.24 ± 0.24	31.71 ± 0.02
P30%-C70% F18/T6	1.34 ± 0.01	3.75 ± 0.01	3.59 ± 0.01	47.79 ± 0.10	23.39 ± 0.02

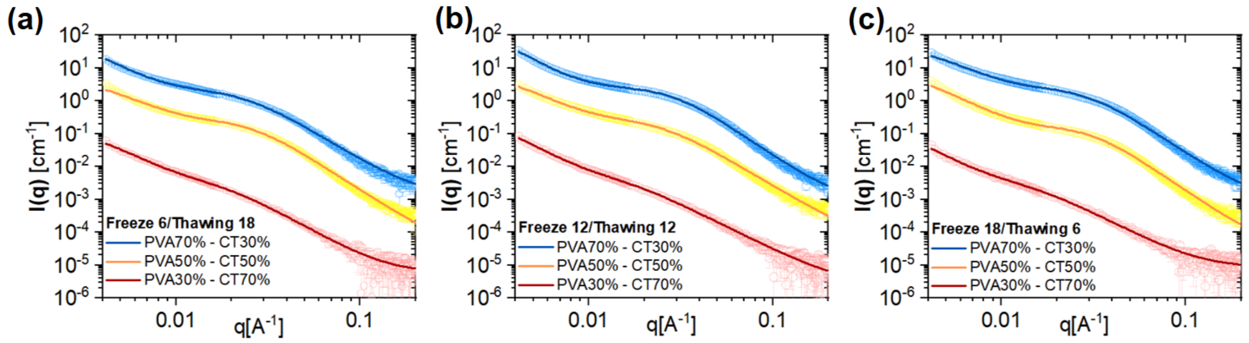

Figure S3. SAXS intensity curves I(q) vs. q for samples with CT Medium-M_w and different PVA/CT, for FT cycles **(a)** F6/T18 and **(b)** F12/T12 and **(c)** F18/T6. For clarity the curves are displaced along the y-axes. Lines are fits to the model of eq. 3 in the main article

Table S2. SAXS parameters obtained from modeling the curves for Medium-M_w of Figure S2 using eq. 3 of the main article.

Sample	Ilor(0)	ζ [nm]	m	Iex(0)	a [nm]
P70%-C30% F6/T18	2.12 ± 0.01	4.24 ± 0.01	3.37 ± 0.01	151.61 ± 0.52	34.58 ± 0.04
P70%-C30% F12/T12	2.50 ± 0.01	3.48 ± 0.01	3.76 ± 0.01	488.51 ± 0.91	42.48 ± 0.02
P70%-C30% F18/T6	2.40 ± 0.01	3.39 ± 0.01	3.70 ± 0.01	72.04 ± 0.15	22.39 ± 0.02
P50%-C50% F6/T18	2.49 ± 0.01	3.81 ± 0.01	3.63 ± 0.01	68.86 ± 0.16	22.89 ± 0.02
P50%-C50% F12/T12	2.72 ± 0.01	4.02 ± 0.01	3.34 ± 0.01	109.20 ± 0.23	25.65 ± 0.02
P50%-C50% F18/T6_3	1.57 ± 0.01	3.19 ± 0.01	3.83 ± 0.02	126.93 ± 0.24	26.29 ± 0.02
P30%-C70% F6/T18	0.38 ± 0.01	5.68 ± 0.02	3.12 ± 0.01	23.91 ± 0.13	27.22 ± 0.05
P30%-C70% F12/T12	0.56 ± 0.01	6.54 ± 0.02	2.81 ± 0.01	77.44 ± 0.35	37.16 ± 0.05
P30%-C70% F18/T6	0.37 ± 0.01	6.95 ± 0.02	2.88 ± 0.01	38.03 ± 0.37	38.15 ± 0.11

3. SEM images, confocal stacks and porosity renderings

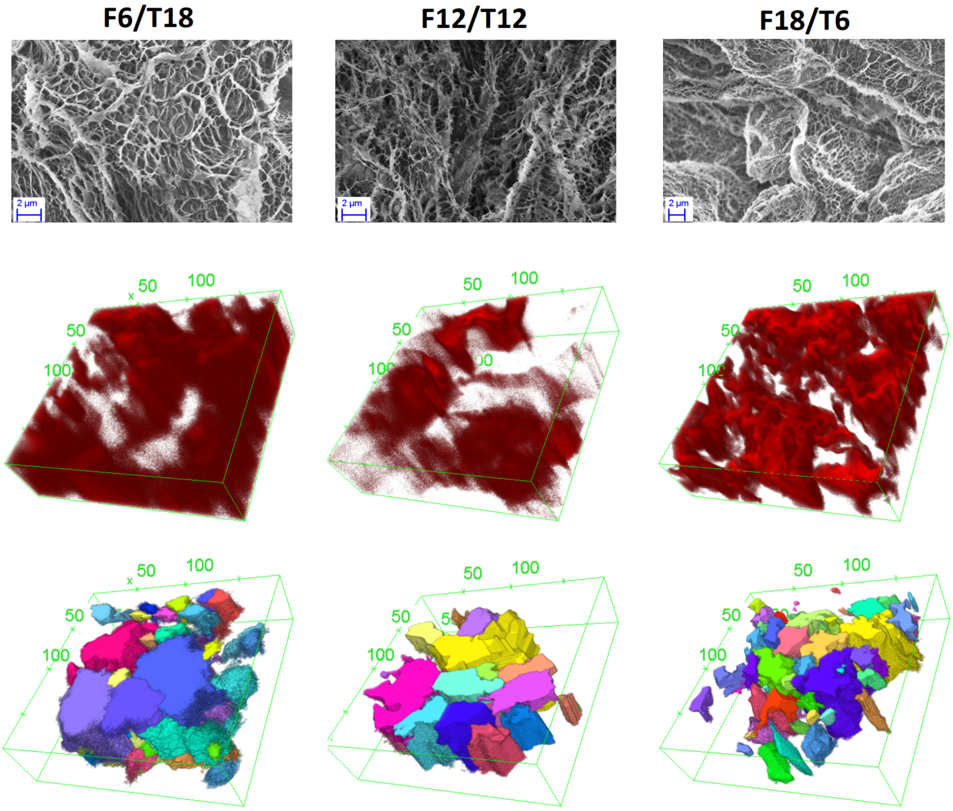

Figure S4. Images correspond to PVA30%- CT70% and different Freeze/Thawing times. **Top** SEM images, scale bar 2µm. **Middle** 3D confocal microscopy images. **Bottom** Hydrogel pore reconstruction using MorpholibJ, units are in µm.

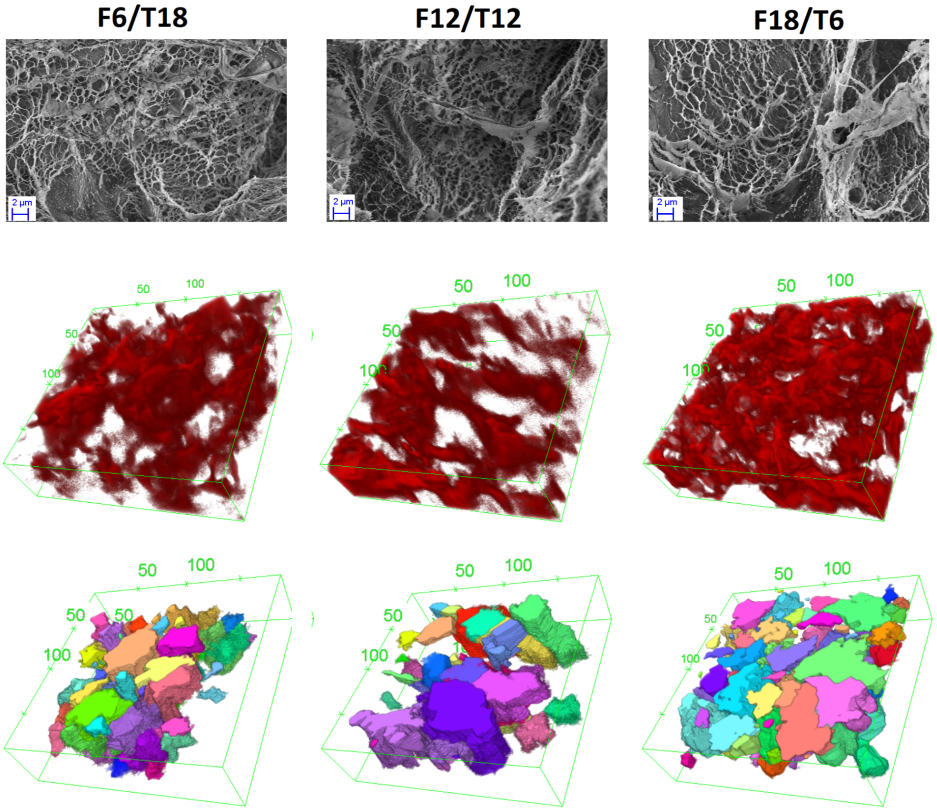

Figure S5. Images correspond to PVA50%- CT50% and different Freeze/Thawing times. **Top** SEM images, scale bar 2µm. **Middle** 3D confocal microscopy images. **Bottom** Hydrogel pore reconstruction using MorpholibJ, units are in µm.

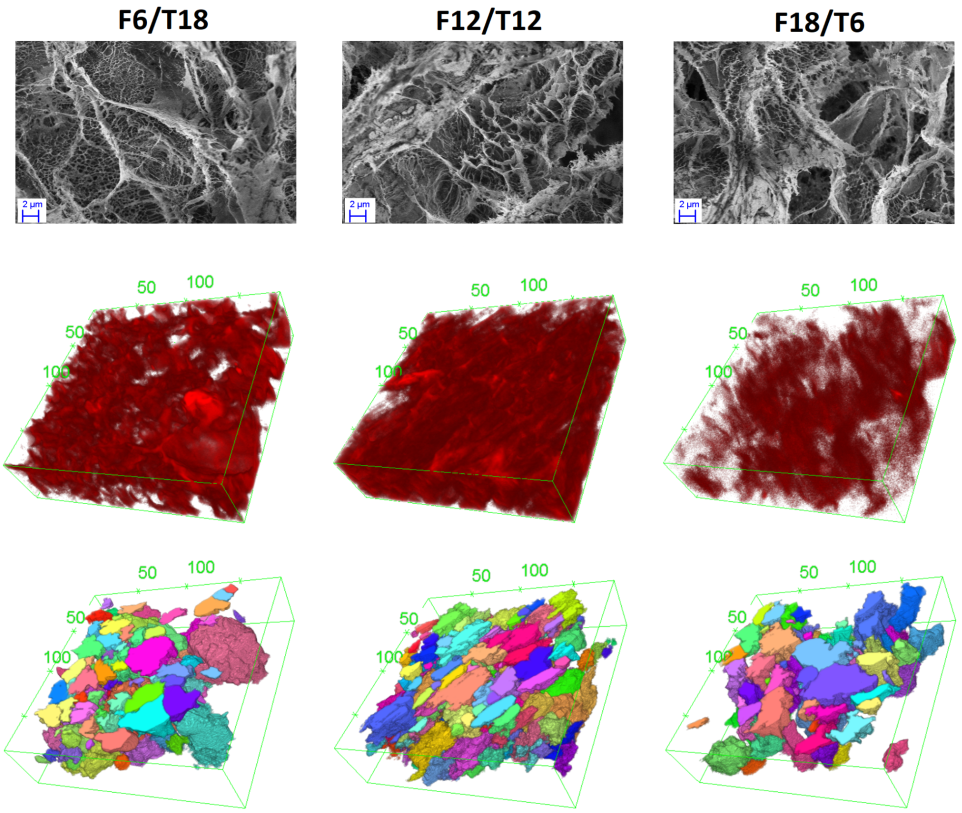

Figure S6. Images correspond to PVA70%- CT30% and different Freeze/Thawing times. **Top** SEM images, scale bar 2µm. **Middle** 3D confocal microscopy images. **Bottom** Hydrogel pore reconstruction using MorpholibJ, units are in µm.

4. 2D porosity parameters and pore volume and sphericity distributions:

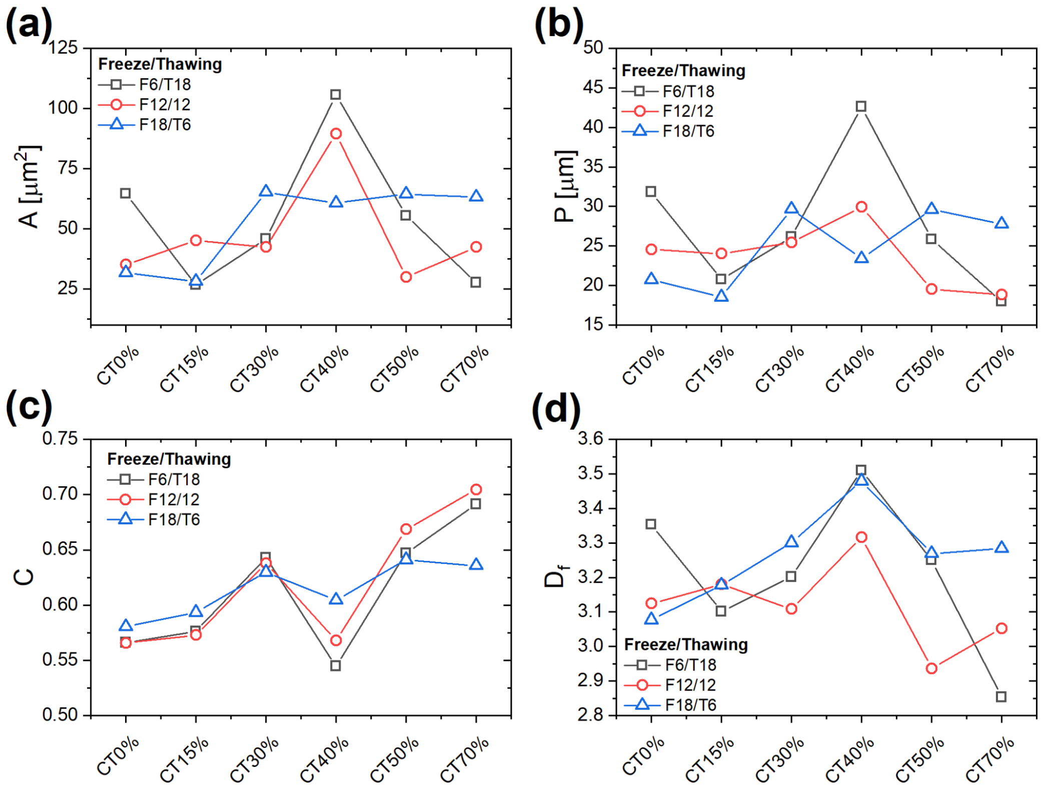

Figure S7. 2D porosity parameters as a function of CT content and for different FT times. **(a)** Average pore area (A) . **(b)** Average pore perimeter (P). **(c)** Circularity (C) parameter calculated as C= $4\pi(A/P^2)$. **(d)** Fractal dimension of pores Df, calculated using the relationship; $A \propto P^{2/D_f}$.

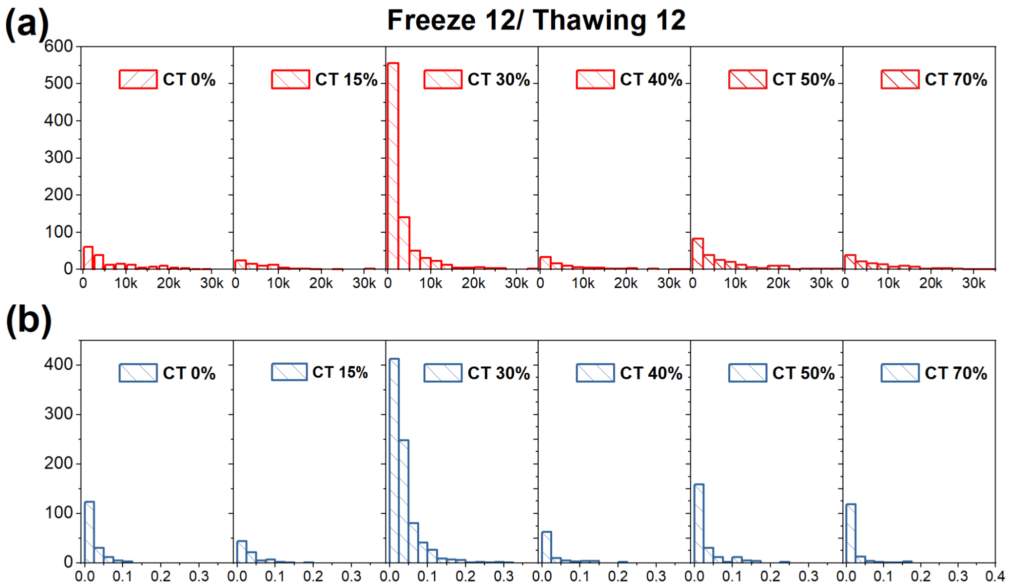

Figure S8. (a) Average pore volume distribution and **(b)** Sphericity distribution for samples obtained using a F12/T12 cycle and different CT content.

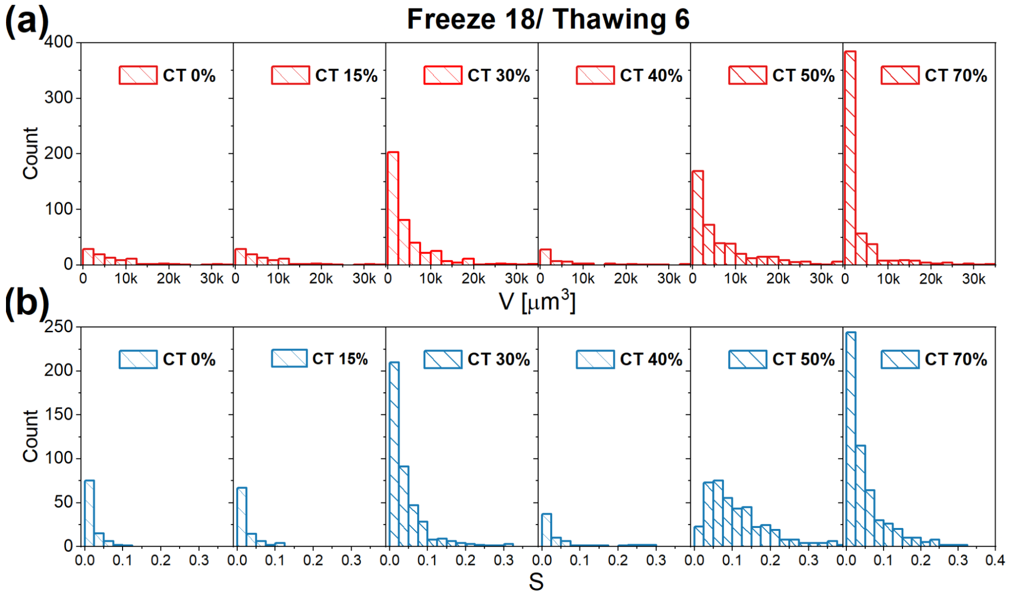

Figure S9. (a) Average pore volume distribution and **(b)** Sphericity distribution for samples obtained using a F18/T6 cycle and different CT content.

5. Rheological data

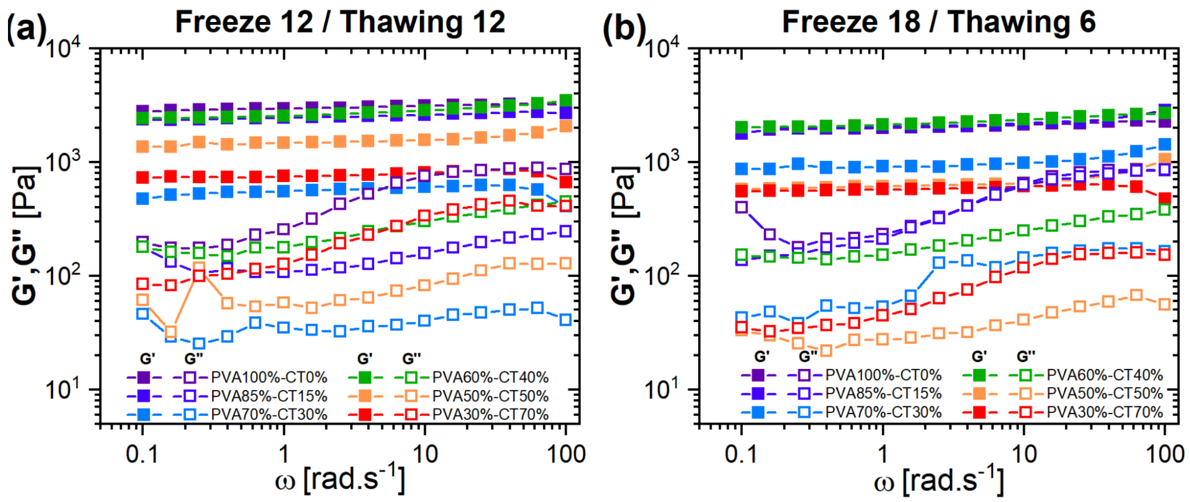

Figure S10. Hydrogel viscoelastic moduli, storage-G′(ω) and loss-G″(ω), for different PVA/CT contents and diverse freeze-thawing times **(a)** F12/T12 and **(b)** F18/T6, as a function of oscillation frequency ω.
